# Infections as a Cause of Preterm Birth: Amniotic Fluid Sludge—An Ultrasound Marker for Intra-Amniotic Infections and a Risk Factor for Preterm Birth

**DOI:** 10.3390/diagnostics15162080

**Published:** 2025-08-19

**Authors:** Mariam Al Baloushi, Badreldeen Ahmed, Justin C. Konje

**Affiliations:** 1 Women’s Wellness Research Centre, Hamad Medical Corporation, Doha 3050, Qatar; 2 Department of Obstetrics and Gynaecology, Qatar University, Doha 2713, Qatar; probadreldeen@hotmal.com; 3 Feto Maternal Centre Al Markhiya, Doha 34181, Qatar; 4 Department of Obstetrics and Gynecology, Weill Cornell Medicine, Doha 24144, Qatar; 5 Department of Health Sciences, University of Leicester, P.O. Box 7717, Leicester LE2 7LX, UK

**Keywords:** ultrasound scan preterm birth, infection/inflammation, antibiotic fluid sludge, antibiotic

## Abstract

Preterm labour (PTL) affects about 11% of all deliveries world-wide. It is a major cause of perinatal morbidity and mortality. Although the precise cause is unknown in about 50% of cases, infections are thought to be a major contributing factor. These infections are more common in earlier preterm deliveries. While some women with these infections will manifest the classical features of fever, tachycardia (maternal and/or fetal), leucocytosis, raised biomarkers of infections, and uterine tenderness/irritation, others will be asymptomatic. Some of the women may develop a short/dilating cervix without any obvious contractions. Identifying such women is potentially challenging. Evidence has shown that a condensation of echogenic particles just above the cervix—amniotic fluid (AF) sludge, identified by ultrasound—is a marker for microbial invasion of the amniotic cavity (MIAC) and preterm birth (PTB) in both asymptomatic and symptomatic women (including those with a short or normal cervix). Those with a short cervix with AF sludge have a significantly greater risk of progression to PTB. Treatment with antibiotics has been shown in some but not all case series to result in a resolution of the sludge and either a delay or prevention of PTB. The widely varied results from treatment could be related to the antibiotics used and the route of administration. The use of the parenteral combination of clindamycin, a cephalosporin, and metronidazole has been shown to be more effective compared to azithromycin. Here we review the literature on the relationship between the sludge and PTB and conclude (1) that the AF sludge is an ultrasound marker of MIAC and PTL and (2) that following its diagnosis, appropriate counselling should be offered and the triple antibiotic combination offered. We suggest that randomised trials should be undertaken to determine the most efficacious antibiotic combination.

## 1. Introduction

Preterm birth (PTB), defined as delivery before 37 completed weeks of gestation, is the leading cause of severe neonatal morbidity and mortality world-wide and accounts for about 50% of neonatal deaths [1,2]. Of the estimated 15 million babies born preterm globally, 5% are before 28 weeks, 10% are between 28 and 32 weeks, and 84% are between 32 and 36 weeks [3]. It is estimated that of all the deaths in the first 5 years of life, about a million (18%) are in children born preterm [4]. The overall PTB rate is about 11%, but this varies from 4–20% depending on the WHO region/country, with the highest rates being in low-income countries. [3,5,6]

Although PTB has been defined by gestational age, there is increasingly a drive to change its taxonomy to phenotypes that incorporate variables such as risk factors, causal conditions, clinical presentation, nutritional status, laboratory features, and mechanisms of action [7,8]. The traditional definition of PTB as a single clinical entity based on gestational age alone fails to acknowledge its syndromic characteristics [9,10]. Furthermore, there has also been a drive to incorporate into this new approach taxonomy biomarkers that reflect maternal vascular malperfusion (e.g., placental growth factor—P1GF, soluble fms-like tyrosine-kinase 1—sFlt-1, and pregnancy-associated plasma protein-A—PAPP-A) [11,12,13]. It must, however, still be acknowledged that setting an upper limit of 36^+6^ weeks’ gestation for defining PTB, whilst it is arbitrary and there is no strong biological reason for doing so, remains a crucial summary marker for neonatal outcomes because it reflects organ immaturity, i.e., increased risk of death, as well as short- and long-term complications [14].

The exact cause of spontaneous preterm birth (SPTB) is unknown in as many as 50% of cases, and it is generally accepted that it is multifactorial in a large number of cases [3,15]. Infections appear to be a common pathway in approximately 25% of cases (and it is probably a common pathway in a much higher percentage in deliveries before 23 weeks, at 79%, with the percentage declining to about 11% for deliveries between 28 and 34 weeks) [16,17]. The presentation of these infections varies from obvious features of chorioamnionitis (such as fever, uterine tenderness, maternal leucocytosis and tachycardia, fetal tachycardia, and foul-smelling/malodourous vaginal discharge) to no obvious clinical features. Confirmation of microbial invasion of the amniotic cavity (MIAC) has traditionally been either from cultures of amniotic fluid and tissues and/or histologically on examination of fetal membranes, the umbilical cord (funisitis), and the decidua [18,19,20]. Several studies have demonstrated the presence of infections in the amniotic fluid, fetal membranes, and cervico-vaginal secretions in women presenting in spontaneous preterm labour (SPTL) [19,20,21]. In those with a short cervix, the evidence is overwhelming [22,23], with typical organisms identified including *Ureaplasma urealyticum*, *Gardnerella vaginalis*, *Candida albicans*, and *Fusibacterium* spp. [24,25]. For diagnosing intra-amniotic infections, opinions vary on whether this should be by amniocentesis routinely performed on all those presenting in SPTL [21] or only by screening the vagina/cervix for infections. Tests on the amniotic fluid, when obtained, include (a) those able to generate rapid results (such as the quantification of glucose, interleukin-6, and MMP-8 levels, white blood cells, and microscopy that could guide management) and (b) cultures that may require time to generate results. The increasing use of molecular biology diagnostic approaches (such as the multiplex PCR test) has improved the rapidity of diagnosis and increased the isolation of micro-organisms that would otherwise not have been identified from routine cultures [25,26].

While the evidence is robust for the role of infections in those in SPTL with or without a short cervix [24], this is less so in those who are asymptomatic and with a normal cervix, who may eventually progress to deliver preterm. Some of these women may have subclinical chorioamnionitis. Early identification of these women and timely institution of interventions (such as antibiotics) may reduce the risk of progression to preterm labour, not only in the symptomatic but also in the asymptomatic women. A key challenge for clinicians is whether there are features that may be indicative of a possible infection prior to shortening and dilatation of the cervix or contractions in this population. An ideal setup will be one where these women at risk are identified and timely interventions to interrupt the process of preterm labour are instituted. This can only be achieved with reliable and specific markers of the causes of SPTB. Ultrasound scans can identify a population with cervical changes and allow/enable interruptions (e.g., cervical cerclage) that have indeed been shown to reduce/delay SPTB. Another possible ultrasound marker, for MIAC—a process that has been shown to predispose mothers to SPTL—is the amniotic fluid sludge [27]. This review brings together the evidence for considering AF sludge as a marker for MIAC and the need to treat this with antibiotics.

## 2. Amniotic Fluid (AF) Sludge and Its Constitution

Particulate materials in the amniotic cavity are a common ultrasound scan finding. They are in general evenly distributed in the amniotic cavity and are thought to represent desquamated fetal cells, vernix caseosa, meconium, and, in some cases, blood [28,29,30,31]. This can also be pathological, where there is excessive desquamation, as in the case of congenital ichthyosis [32]. These particulate materials have been reported in about 4% of scans performed between the first and second trimesters [33,34], a percentage that rises to about 88% by 35 weeks [35]. AF sludge, on the other hand, is a dense aggregate of highly echogenic material accumulating above the cervix, which is present in about 1% of uncomplicated term pregnancies [19], with the percentage rising to about 23.5% in high-risk populations (spontaneous preterm labouring women with intact membranes) [36]. Espinoza and colleagues proposed this term to describe this free-floating hyperechogenic material and associated it with an increased risk of SPTB [27].

The location of the AF sludge and its association with microbial invasion of the amniotic cavity is highly suggestive of an infective/inflammatory process (involved in its formation) [37]. Micro-organisms may reach the amniotic cavity by breaching the membranes (when they ascend from the lower genital tract) or transplacentally, reaching through a haematogenous spread [38]. On reaching the amniotic cavity, the micro-organisms provoke an inflammatory response in the epithelial cells of the fetal skin, the membranes, and the umbilical cord [39]. As a result, there is a significant increase in pro-inflammatory cytokines (typically IL-1, IL-6, and IL-8 and TNF-α), prostaglandins, chemokines, and an increased expression of matrix metalloproteinases [37,39]. The increased expression of cytokines/chemokines in the amniotic cavity stimulates the migration of neutrophils across the decidua and the chorioamniotic membrane into the amniotic cavity, resulting in an increased white cell population and enhanced anti-microbial activity [37,39,40]. The facts that some studies have failed to isolate micro-organisms and that the AF sludge has also been found in pregnancies that progress to term suggest that the infective pathogenesis may not be applicable in all cases. Some have suggested that intra-amniotic inflammation can be a result of exposure to “danger signals” produced by cells undergoing stress/damage or death [41,42,43,44]. This is supported by the fact that sterile inflammation is more common in women presenting with SPTL and preterm premature rupture of fetal membranes (PPROM) compared to the classical microbiological inflammation [41,42,43,44]. Of note is also the fact that conventional cultures are only able to accurately identify infection in a proportion of cases. The increasing use of modern approaches to identify infectious organisms (PCR techniques) has increased isolation rates [26,27].

In an interesting report, Romero et al. not only performed an amniocentesis from the sludge but were able to physically observe it in vitro. The sludge was described as resembling pus on naked-eye examination. Gram staining from this and other studies has shown the presence of a variety of organisms (*Mycoplasma hominis*, *Streptococcus mutans*, and *Aspergillus flavus*). The key question, therefore, is what is the precise composition of this AF sludge? It has been postulated that progressive infection induces an intense inflammatory response and that the inflammatory cells from this response (neutrophils) combine with the micro-organisms to form the particulate material that is visible as the AF sludge [44].

When micro-organisms breach the feto-maternal barrier, the consequence depends on how large the dose of micro-organisms reaching the fetal membranes is—with the risk of infection/inflammation greatest with the highest micro-organism load [44]. These micro-organisms (bacteria) can exist in one of two forms—singly (in the planktonic form) or organised in biofilms or both [44]. Following invasion of the amniotic cavity, bacteria change their phenotype to protect themselves from the host response (which includes the generation of inflammatory cells (neutrophils and monocytes) and the production of anti-microbial peptides and other mediators which can kill or injure the bacteria [44]. The bacteria achieve this by aggregating themselves in building-like structures known as biofilms. These biofilms make the micro-organisms resistant to attack by the macrophages, natural or synthetic antibiotics, and anti-inflammatory mediators [37,38,39,40]. The bacteria in these biofilms are less likely to elicit an inflammatory response [28]. The formation of these intra-amniotic biofilms would partly explain why the intra-amniotic infection tends to be chronic and perhaps why they are difficult to treat, as these biofilms are relatively resistant to antibiotic treatment [45,46,47,48,49]. The balance between the proportion of bacteria that are in the planktonic form and those in the biofilms likely determines the course of the infection and the likelihood of positive cultures from amniotic fluid sampling. Planktonic bacteria are more likely to be cultured positive as opposed to those in the biofilm [44,47]. In some cases, no organisms have been isolated.

The concept of sterile inflammation, as has been found in some cases with amniotic fluid sludge, is supported by the recent findings by Wu et al. [50]. It would appear that there may be complex potential links between placental dysfunction and inflammation [51]. Both infection and sterile inflammation mechanisms of immune origin (including maternal antifetal rejection) may lead to infiltration of the placenta by lymphocytes, plasma cells, and/or macrophages, leading to chronic placental inflammatory lesions, which may be responsible for both abnormal placental function and perhaps alterations in maternal white blood cell distributions, as shown in the study by Wu et al. [50]. This could perhaps explain the failure to identify organisms in some cases of amniotic fluid sludge, but underscores the potential mechanisms by which these women progress to PTB.

## 3. Imaging for AF Sludge

**Figure 1:**
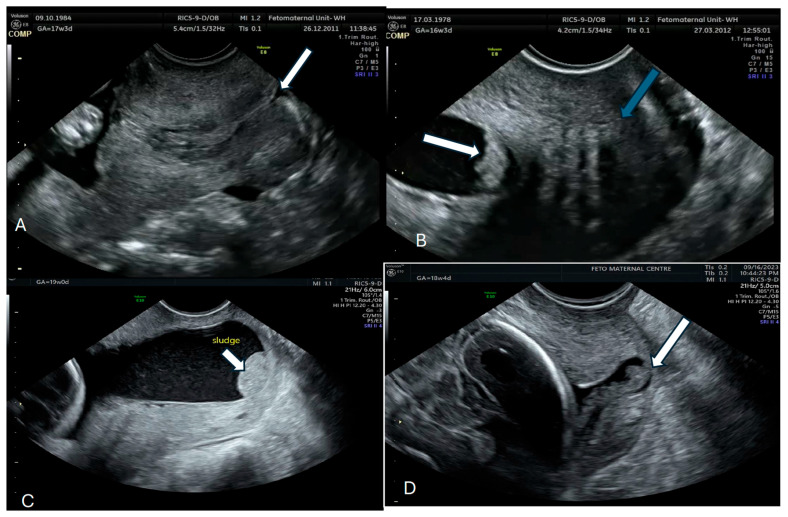
Normal cervix with no sludge arrow (**A**); Amniotic sludge (arrow with normal cervix-green arrow) (**B**,**C**) Sludge (white arrow) and Sludge with a dilated cervix-white arrow (**D**).

The best approach to identifying AF sludge is with a transvaginal ultrasound scan with the woman lying in the lithotomy position after emptying her bladder. The probe should be placed in the anterior fornix and adjusted to obtain a sagittal view of the entire cervical length. The length of the cervix should be measured preferably thrice and the average obtained. AF sludge is diagnosed from the presence of a dense aggregate of highly echogenic particulate matter in close proximity to the internal cervical os [27,52]. This material may scatter with fetal movements or abdominal pressure, but should re-accumulate within a few seconds. Where facilities are available, VOCAL^TM^ software (manufactured by GE Healthcare Technologies, Inc., Chicago, IL, USA) can be used to measure the volume of the AF sludge. We recommend that at least 3 min be spent assessing the sludge because of the dynamic nature of this marker. Figure 1 shows the AF sludge in four of our patients.

## 4. AF Sludge and Intra-Amniotic Infections

An investigation of AF sludge aspirated by amniocentesis showed this to be positive for the micro-organisms *Streptococcus mutans*, *Mycoplasma hominis*, *Ureplasma urealyticum*, and *Aspergillus flavus* [53]. Interestingly, in a study by Yoneda and colleagues of women presenting in preterm labour at 20–29 weeks, [54] using polymerase chain reaction (PCR), the AF “sludge” was present in 18.1% (19/105) of patients. However, there was a similar positive micro-organism rate in the women with sludge and those without (31.6% versus 38.4%), but a significantly higher level of amniotic fluid interleukin-8 (15.2 ng/mL vs. 5.8 ng/mL; *p* = 0.005) and a higher frequency of histological chorioamnionitis in those with sludge (52.6% vs. 23.3%; *p* = 0.01). In another study of 25 women with AF sludge, examination of amniotic fluid collected after amniotomy showed that it was frequently associated with an inflammatory process [55]. Gill et al., in a cohort of 62 women with a short cervix and AF sludge (AFS) who had undergone amniocentesis, showed the rate of intra-amniotic inflammation to be 31.4% vs. 3.7% in those who delivered <32 weeks compared to those who delivered after, and furthermore, histological chorioamnionitis was significantly more common in the former group (75% vs. 32%) [56]. Interleukin-8 was shown to have the highest sensitivity and specificity for intra-amniotic inflammation and histological chorioamnionitis in this cohort. In an earlier study, Kusanovic and colleagues [36] found a higher positive culture rate in those with sludge (33.3% vs. 2.5%; *p* = 0.003), and a higher frequency of histological chorioamnionitis (77.8% vs. 19%; *p* < 0.001). The findings of histological chorioamnionitis and funisitis were in four cases presenting with cervical insufficiency and AF sludge [57]. Despite all these studies showing a high frequency of infection/inflammation in the presence of AF sludge, Ventura et al., in a study of 16 cases, failed to show any difference in these parameters, although the women with AF sludge delivered earlier [58]. Taken together, these case series appear to suggest that AF sludge is a proxy or indeed a marker for microbial invasion of the amniotic cavity, which in some cases may manifest as intra-amniotic infection and/or inflammation and, therefore, is a risk factor for PTB. There is a need for more studies to investigate just how good a marker of preterm birth AF sludge is. Table 1 summarises the studies that have investigated the association between AF sludge and intra-amniotic infection/inflammation [27,36,54,57,58,59,60].

## 5. AF Sludge and an Ultrasound Marker for Spontaneous Preterm Labour?

Since intra-amniotic infections are generally associated with SPTB, and AF sludge is thought to represent MIAC, the key question is whether AF sludge could be an ultrasound marker of PTB. Identification of AF sludge in the first half of pregnancy has been shown to be associated with inflammation/infection, while in late pregnancy, it is thought to reflect maturation of the fetus (i.e., dominated by vernix caseosa, fetal squames, and meconium) [28,29,61,62,63]. Espinoza et al. [27], in a retrospective study of 84 women (19, or 22.4%) with intact membranes and spontaneous preterm labour, and 298 (1% with AF sludge) at term with intact membranes, concluded that the presence of AF “sludge” in the spontaneous preterm labour and intact membranes group was a risk factor for MIAC, histological chorioamnionitis, and impending spontaneous preterm delivery. Several other case series (mostly retrospective) have not only shown that those with AF sludge are at greater risk of SPTL but that this risk is much greater in those with a short cervix. In a large retrospective study of 281 asymptomatic women who underwent cervical length measurement and screening for AF sludge, Kusanovic et al. [36] showed AF sludge to be present in 66 cases. The shorter the cervical length, the higher the presence of AF sludge. The SPTB rate was higher in those with AF sludge, and this significant difference was maintained for PTB < 28, <32, and <35 weeks. The combination of a cervical length of <25 mm and the presence of AF sludge conferred odds ratios of 14.8 and 9.9 for spontaneous delivery at <28 and <32 weeks, respectively [36]. Although some studies have concluded that the presence of AF sludge is an independent risk factor for the occurrence of SPTB, these observations have not been universal. There has, to the best of our knowledge, been only one prospective study [59]. Table 2 is a summary of the reports on the association between amniotic fluid sludge and SPTB [27,33,34,36,52,58,64,65,66,67].

## 6. Will Treatment Improve Outcomes?

Since there is strong evidence associating AF sludge with infection/inflammation, it follows that treatment with antibiotics may reduce the risk of PTB. This was the basis of several studies that have investigated the effect of antibiotics in women at risk of SPTB based on the ultrasound finding of AF sludge. The results from various studies on the efficacy of antibiotics in this regard have been inconclusive. These studies (reviewed below) include case reports and retrospective and prospective studies. The first report of the use of antibiotics in women with AF sludge was reported by Himaya et al. [59]. They describe a woman diagnosed with the sludge at 15^+6^ weeks with a cervical length of 33 mm treated with an intravenous ampicillin–gentamicin and oral azithromycin combination following an amniocentesis and culture of *Staphylococcus warneri* at 22 weeks. Following treatment, a second amniocentesis was performed, and the culture was negative. She progressed to deliver at term. A historical controlled observational study by Hatanaka et al. [68] was reported in which women with AF sludge diagnosed before 2012 and who were not treated were compared with those diagnosed after 2012–2015 and treated with antibiotics. The women were divided into two groups—those at low risk were given oral clindamycin (300 mg every 6 h) and cephalexin (500 mg every 6 h) for 7 days, while the high-risk group was given intravenous clindamycin (600 mg every 8 h) and cefazolin (1 g every 8 h) for 5 days followed by oral treatment for 5 days. They showed that there was a reduction in the PTB rate in the treated group compared to the untreated group (13.2% vs. 38.5%). Cuff et al. [67], in a retrospective cohort study of 97 women with sludge, compared outcomes between those who had either been given oral azithromycin 500 mg on day 1 followed by 250 mg orally for 4 days or moxifloxacin 400 mg orally taken daily for 5 days and those who had not been treated. They showed that sonographic resolution of the AF sludge occurred in 34% of those who had been treated and 43% of those who had not—a difference that was not statistically significant. Furthermore, there were no differences in the rates of SPTB in both groups. Pustotina [69] undertook a prospective study of 29 women with AF sludge (some with a short cervix and symptomatic) in which they offered vaginal clindamycin and other combinations (cefoperazone, clavulonate + amoxicillin, and, in some cases, and in some, combined with indomethacin, progesterone, and IV sulbactam) and showed that antibiotic treatment eliminated the AF sludge and prevented SPTB in all the cases. Jin et al. [70], in a retrospective study of 58 women with a sludge diagnosed at 15–23 weeks and treated with a combination of IV ceftriaxone, clarithromycin, and metronidazole, showed a lower level of preterm birth following treatment, with disappearance of the sludge in 51.7% of cases. More recently, Giles et al. [71] reported on a retrospective cohort of women with AF sludge who were treated and compared the outcomes to those not treated. The antibiotic used was azithromycin. Interestingly, the overall spontaneous preterm birth rate was higher in the treatment group, but there were no differences in neonatal morbidity. Table 3 summarises all these studies [67,68,69,70,71,72,73]. Two meta-analyses [74,75] and a review of the literature by Luca et al. [76] concluded that while AF sludge is a marker of preterm birth, there are no robust data on the benefit of antibiotics in this group. More recently, there have been reports on cases where AF sludge was identified and antibiotics administered and followed until it disappeared, and the pregnancy progressed to term [73].

A major factor that has been suggested as a potential confounder responsible for the variable results is the different antibiotic regimens used and the routes of their administration. Additionally, the studies have been very heterogenous, varying from those on asymptomatic low-risk/high-risk women to those on women with symptoms (i.e., presenting with uterine activity/contractions) or a combination of both. The two studies that showed no difference in treatment used azithromycin as the main antibiotic compared to the others that showed a difference (all of which used a variety of antibiotics, including intravenous clindamycin and a cephalosporin). From these data, it would seem that the biofilm is less likely to be penetrated by antibiotics administered orally as these may not achieve high levels in the blood. It would therefore be reasonable to recommend that these women are offered intravenous antibiotics. We have treated a number of cases in our unit (*n* = 21) over the last 18 months with AF sludge with a combination of intravenous clindamycin, metronidazole, and ceftriaxone (given for one week) and in 14 out of the 21 (67%) cases, the AF sludge resolved, and pregnancies progressed to term, or delivery was delayed by an average of 2 weeks in those who delivered preterm. We believe that considering how common this finding is in women at risk of SPTL, there should be randomised controlled trials on the efficacy of intravenous clindamycin combined with a cephalosporin and metronidazole to determine if such a regimen will reduce the risk of SPTB and therefore neonatal morbidity and mortality. This is the combination that appears to be most effective from the case series and covers most of the spectrum of organisms that have been isolated from the various studies. Clindamycin, for example, covers Gram-positive organisms and many anaerobes, including most strains of *B.* *fragilis* and β-lactam—resistant strains of *S. pneumoniae* and *Staphylococcus*. Metronidazole, on the other hand, is used because it is also effective against anaerobic bacteria and certain protozoa. Specifically, it is active against various anaerobic bacteria such as Bacteroides, Fusobacterium, Clostridium, *Gardnerella vaginalis*, Prevotella, Porphyromonas, and *Peptostreptococcus species*. Ceftriaxone is effective against Gram-positive (Streptococcus and staphylococcus species) and Gram-negative bacteria (especially Enterobacteriaceae, *E.* *coli*, and Klebsiella). We recommend this combination because most studies showed it to be the most effective, covering most of the pathogens that have been isolated in the amniotic fluid (Table 1). There are no studies that show a close relationship between cervical pathogens and the pathogens from the amniotic fluid sludge, hence the recommendation to use a broad-spectrum combination. One study investigated [77] the cervical inflammatory markers (IL-8) and showed them to be higher in those with sludge, but no organisms were cultured.

Finally, it could be argued that leaving the fetus in utero with MIAC would increase morbidity. The fact that the antibiotics led to resolution of the AF sludge in many cases and that the case series reviewed have not reported increased morbidity in the neonates (if anything, delaying birth was associated with better outcomes) is reassuring in this context. Prior to commencing women on broad spectrum antibiotics, the potential of bacteria resistance must be discussed.

## 7. Conclusions

There is no doubt that intra-amniotic infections are central to a significant proportion of preterm births. Some of these manifest as obvious clinical infections, and in most cases, with uterine contractions and a short/dilated cervix. In some cases, however, the infections may be subclinical/asymptomatic. Identifying such cases is challenging, but the presence of AF sludge has been shown in case series to be a marker of such infections. The evidence linking amniotic sludge with SPTB, although predominantly from case series, is moderately robust, although more data are required. What is uncertain is whether treatment with antibiotics does indeed lead to the resolution of AF sludge and prevention/delay of PTB. The potency of the antibiotics depends on the type of antibiotics and how they are administered. The data reviewed here suggests that oral azithromycin is not effective, while a combination of parenteral ceftriaxone, metronidazole, and clindamycin appears to be effective. While we feel there is enough to suggest treating these women with this combination, there is a need for randomised controlled trials on the efficacy of this regimen to generate robust evidence. Such studies ideally should include other factors such as maternal characteristics and inflammatory biomarkers (e.g., IL-1 and -6, TNF-α, IL-8, and MMP-9) in amniotic fluid, plasma, and vaginal secretions [67] that increase the risk of preterm birth to allow for a better generation of algorithms for the prevention of PTB. Until such studies are undertaken, clinicians must continue to counsel women diagnosed with AF sludge on the pros and cons of antibiotics and the limited data on efficacy.

## Figures and Tables

**Table 1:** Summary of studies that have investigated intra-amniotic infection/inflammation in women with amniotic sludge.

Study Details (Authors and Year)	Type of Study	Population Studied and Gestation at Study	Method of Investigation	Principal Findings	Organisms Isolated from Culture/PCR
Espinoza et al., 2005 [27]	Retrospective	84 women in preterm labour at 20–35 weeks and 298 in term labour	Amniocentesis of the preterm group (*n* = 84) for culture. 19 had sludge and 65 did not. Histological examination of the amniochorion and placenta.	Histological chorioamnionitis in those with and without sludge. 77.8% (14/18) vs. 19% (11/58); *p* < 0.001 and positive AF culture 33.3% (6/18) vs. 2.5% (1/40); *p* = 0.003.	*Ureplasma urealyticum*, *Fusobacterium nucleatum*, *Candida albicans*, *Peptostreptococcus* spp., *Gardnerella vaginalis*
Kusanovic et al., 2007 [36]	Retrospective case control	281 asymptomatic women at 13–29 weeks with a short cervix	66 had sludge and 215 did not. Amniocentesis performed on 51 (23 with sludge and 28 without) for AF culture and WBC in AF of >50 cells/mm^3^. Histology of membranes and cord.	MIAC rates of 21.7% (5/23) in AF sludge vs. 0% (0/28) in non-sludge group and 27.3% (6/23) vs. 3.6% (1/28) for intra-amniotic inflammation.	*Urealasma urealyticum*, *Staphylococcus aureus*, and *Fusobacterium nucleatum*
Himaya et al., 2011 [59]	Prospective	310 women undergoing karyotyping by amniocentesis at 14–24 weeks	Quantification of amniotic fluid concentration of MMP-8 (MMP-8), glucose, and lactate from 310 women (94 with free-floating particles, 19 with dense amniotic sludge, and 200 with no particles/no sludge). CL normal in all cases except 1 with a CL of <15 mm.	No significant differences in MMP-8, lactate, and glucose in the groups. No differences in markers of MIAC in all groups. Woman with CL < 15 mm had higher MMP-8, and lower glucose. 2 women who delivered <32 weeks had higher mean lactate.	*Staphylococcus warneri* in one case
Ventura et al., 2011 [58]	Retrospective case control	58 women in preterm labour at 22–34 weeks	Two groups—16 with sludge and 42 without. Histological examination. Histological chorioamnionitis was based on the presence of inflammatory cells in the chorionic plate and/or chorioamniotic membranes.	No difference in histological chorioamnionitis between those with and without sludge (18.8% vs. 14.3; *p* = 0.067).	Organisms not characterised
Paules et al., 2016 [57]	Case report at 21–24 weeks	4 cases of cervical weakness and bulging membranes with amniotic fluid sludge	Amniocentesis in 3/4 cases (one of the cases refused amniocentesis).	All had histological chorioamnionitis and 2 had funisitis.	*Fusobacterium nucleatum* (in 2/3 cases)
Pedregosa et al., 2017 [60]	Retrospective and prospective	Asymptomatic/symptomatic women with a short CL <25 mm at 16–32 weeks	Amniocentesis in 15 cases—12 with sludge. PCR, culture, Gram staining, and WBC and glucose levels. Microbiological study of placenta, membranes, and umbilical cord.	From 15 amnios, 8 had MIAC and 6 had sterile inflammation (without any isolated organism) and 1 was negative. 10 positive cultures of placenta, membranes, and cord.	Genital mycoplasma (*Ureaplasma urealyticium, Mycomplasma hominis*—most common organisms)
Yoneda et al., 2018 [54]	Retrospective	105 women in preterm labour at 20–29 weeks	Amniocentesis from 105 women in preterm labour (19 with sludge and 86 without) for culture, PCR (positive AF cultures—examined using a nucleotide sequence-based analysis of bacterial genome DNA or 16 S rRNA metagenomics), and IL-8 and placental histology of placenta.	Women with vs. without sludge PCR—no difference; 31.6% (6/19) vs. 38.4% (33/86); *p* > 0.05. Funisitis 31.6% (6/10) vs. 23.2% (20/86); *p* = 0.447. Histological chorioamnionitis 52.6% (10/19) vs. 23.3; *p* = 0.01. IL-8-15.2 ng/mL vs. 5.8 ng/mL; *p* = 0.005.	*Streptococcus parvum, Streptococcus agalactiae*, *Ureaplasma parvum*, *Flavobacterium succinicans*, *Ureaplasma urealyticum*
Gill et al., 2019 [56]	Cohort	62 asymptomatic women with a short cervix (≤25 mm) at 16–22 weeks	Amniocentesis for concentrations of 33 proteins and histological examination of chorioamnion. Cohort was divided into those who delivered ≤ 32 weeks (*n* = 35) and those who delivered >32 weeks (*n* = 27) and variables were compared (>1.5-fold change in protein concentration considered significant).	Intra-amniotic inflammatory rate higher in <32 week group (31.4% vs. 3.7%; *p* = 0.008); acute histological chorioamnionitis greater (75% vs. 32%; *p* = 0.002); higher mean concentration of 8/13 proteins—with IL-8 showing the highest difference (4.1-fold).	No organisms investigated

CL = cervical length; MIAC—microbial invasion of the amniochorion.

**Table 2:** Summary of studies that have investigated the association between amniotic fluid sludge and preterm labour.

Authors and Year of Study	Type of Study	Population Studied and Gestation of Study	Cervical Assessment	Outcome in Terms of Rates/Risk of Preterm Birth
Espinoza et al., 2005 [27]	Retrospective	Women recruited at 20–35 weeks who went into preterm labour (*n* = 84) and delivered at term; controls (*n* = 298). Sludge present in 19 of the preterm cohort (i.e., 19/84).	CL ≤ 25 mm in all those with sludge (*n* = 19) and 49/65 in those without.	Risk of PTB significantly greater in those with sludge at 48 h and 7 days of delivery from diagnosis, and at 32 and 35 weeks: by 48 h—42.9% vs. 4.4%; by 7 days—71.4% vs. 15.6%; <2 weeks—75.0 vs. 25.8%; <35 weeks—92.9% vs. 37.8%.
Bujold et al., 2006 [33]	Retrospective	89 women at risk of preterm birth recruited for cervical length measurement at 18–32 weeks’ gestation—14 with sludge and 75 without.	CL significantly shorter in those with sludge—34.0 ± 10 mm in those with no sludge vs. 23 ± 11 mm and 16 ± 14 mm in those with light and dense sludge.	Spontaneous PTB in <34 weeks—8/14 (57.1%) vs. 5/75 (6.7%) in those with and without sludge.
Kusanovic et al., 2007 [36]	Retrospective case control	281 patients between 13 and 29 weeks. Sludge *n* = 66, controls *n* = 216.	Cervical length measured and grouped into <5 mm, <15 mm, <25 mm, and >30 mm.	Sludge present in 69% (20/29), 49% (33/68), 35% (49/142), and 12% (12/99), respectively, for CL < 5 mm, <15 mm, <25 mm, and >30 mm. Spontaneous PTB—no sludge vs. sludge—<28 weeks, 9.4%vs 54.3%; <32 weeks, 14.6% vs. 60%; and <35 weeks,19.8% vs. 42.3%. Odds of SPTB if combined sludge and CL < 25 mm—14.8 for delivery <28 weeks and 9.9 for delivery <35 weeks.
Ventura et al., 2011 [58]	Retrospective cohort	58 women with threatened preterm labour at 22–34 weeks—16 with amniotic fluid sludge and 42 without.	Of the 16 with AFS, 75% had CL ≤25 mm and 37.5% had CL ≤ 15 mm.	SPTB greater in those with AFS. 25% vs. 2.4% within 48 h. 37.5% vs. 11.9% within 7 days. 75% vs. 23.9% within 14 days. USS to delivery interval 21.7 ± 30.1 vs. 49.4 ± 137.8 days.
Hatanaka et al., 2016 [52]	Prospective cohort	195 women at 16–26 weeks, 49 with sludge and 146 without.	CL < 25 mm—38.8% (19/49) (sludge) vs. 17.5% (23/146).	Gestational age at delivery in sludge vs. no sludge groups—35.8 ± 5.4 weeks vs. 37.8 ± 3.6 weeks. SPTB rates differed at up to <35 weeks (at <28 weeks—12.2% vs. 3.4%; at <32 weeks—17.1% vs. 5.1%; and at <35 weeks—26.8% vs. 8.5%).
Adanir et al., 2018 [34]	Prospective	92 women at high risk of preterm delivery between 20 and 34 weeks’ gestation—18 with sludge and 74 without.	CL ≤ 25 mm in 8/18 (sludge) vs. 9/74 (no sludge).	SPTB rate of 66.7% (12/18) in those with sludge vs. 27% (20/74) in those without sludge.
Tsunoda et al., 2020 [64]	Retrospective cohort	110 patients at 14–30 weeks—TVS measurement of CL and sludge. 29 with sludge and 81 without.	29 delivered <34 weeks and 51 < 37 weeks. 16/29 and 21/51 had sludge. CL < 20 mm—24/29 vs. 33/51 and <15 mm—17/29 vs. 21/51.	Risk of SPTB increased with the presence of AFS. Odds ratio for delivery <35 weeks—6.44 and <37 weeks—4.46.
Yasuda et al., 2020 [65]	Retrospective	54 women presenting in preterm labour at 22–36^+6^ weeks. Cervical length measured and sludge identified.	Sludge present in 11 cases.	AFS cohort delivered at 28.3 ± 4.5 weeks vs. 31.7 ± 4.3 weeks.
Pahlavan et al., 2022 [66]	Nested case control	110 women who underwent ART in the form of IVF-ET—63 with sludge and 67 without.	CL < 30 mm in control group—10.4% and 28.6% in the study groups.	SPTB prevalence of 23.6% in case and 10.4% in control.
Cuff et al., 2020 [67]	Retrospective cohort	147 women—54 with sludge and 93 without.	Women with sludge more likely to have a short CL (19 mm vs. 14 mm).	Women with AFS + short CL, more likely to have a mid-trimester loss and delivery <24 weeks (RR 3.4; 95%CI 3.4–12–20.3).

SPTB = spontaneous preterm birth; PTB = preterm birth; CL = cervical length; IVF-ET = in vitro fertilisation and embryo transfer; AFS = amniotic fluid sludge.

**Table 3:** Summary of studies that have investigated antibiotics in women with amniotic fluid and its impact on the risk of preterm birth.

Authors and Year of Study	Type of Study	No of Cases Studied Included	Antibiotic Regimen and Duration	Outcome (in Terms of Risk of Preterm Birth)
Fuchs et al., 2015 [72]	Retrospective case control	77 asymptomatic women at 15–32 weeks—63 Rx and 14 untreated. Cervical length measured.	Azithromycin 500 mg on day 1 and then 250 mg IV and oral for 4 days.	Overall SPTB rates—57% (36/63) vs. 29% (4/14); *p* = 0.05 in the treated and untreated groups; PTB < 28 weeks—11.1% vs. 28.6; *p* = 0.1; PTB < 32 weeks—17.5% vs. 42.9%; *p* = 0.07; PTB < 34 weeks—19.1% vs. 57.1%; *p* = 0.006. **Conclusion:** Use of azithromycin reduced the risk of PTB < 34 weeks.
Hatanaka et al., 2019 [68]	Observational historical controlled	Cohort of 86 asymptomatic diagnosed with AFS at 16–26 weeks (divided into high and low risk) and 22 controls with AFS. Cervical length measured.	Two groups. High risk (CL < 25 mm/other risk factors) IV clindamycin + cefazolin for 5 days and then oral for 5 days. Low risk (CL > 25 mm). Clindamycin (oral) + cephalexin for 7 days—low-risk group.	Risk of SPTB < 34 weeks in high-risk group—13.2% vs. 38.5% (*p* = 0.047) in treated vs. untreated groups. No difference in SPTB rate at all gestations in both groups together (i.e., combined high and low risk = treated vs. untreated). **Conclusion:** In high-risk group, antibiotics reduce risk of SPTB < 34 weeks.
Cuff et al., 2020 [67]	Retrospective cohort	97 asymptomatic women with AFS diagnosed at 15–25 weeks—51 treated and 46 untreated. CL measured in both groups.	Mixed treatment. 46 Rx with oral azithromycin × 5 days; 5 Rx with oral moxifloxacin × 5 days.	Overall SPTB rate < 37 weeks—49.5% and 22.7% < 28 weeks. CL measurements same in treated and untreated groups. SPTB < 37 weeks—53% vs. 45.7% in treated vs. untreated (*p* = 0.47). SPTB < 228 weeks—21.6% vs. 19.6% (*p* = 0.81). **Conclusion:** Treatment made no difference in outcome.
Pustotina, 2020 [69]	Prospective	29 asymptomatic women with AFS diagnosed at 14–24 weeks divided into three groups: 14 with CL < 25 mm and symptomatic; 7 with Cl >25 mm and asymptomatic; 8 with CL > 25 mm.	All 29 received vaginal clindamycin suppositories and 16—IV cefoperazone/sulbactam; 8—oral amoxicillin/clavulanate. IV butoconazole to 18. Progesterone and indomethacin given to all those with CL < 25 mm.	Intravenous antibiotics prevented SPTB in all women with CL > 25 mm and asymptotic women with CL < 25 mm and in 70% of those with symptoms and CL < 25 mm. **Conclusion:** Intravenous antibiotics delayed delivery or prevented SPTB.
Jin et al., 2021 [70]	Retrospective cohort	58 women at 15–32 weeks; symptomatic women with intact membranes and AFS.	IV ceftriaxone 1 g daily, clarithromycin 500 mg, BD orally, and metronidazole 500 mg tds—all for 4 weeks.	AFS disappeared in 30/58 (51.7%). USS to delivery interval—67.7 + −35.7 days vs. 28.4 + −35.7 in those without AFS and with persisting AFS after treatment. SPTB <28, <32, and <34 weeks was greater in the persistent group. **Conclusion:** Antibiotics may cause AFS to disappear in women presenting in PTL and this is associated with improved outcomes.
Giles et al., 2023 [71]	Retrospective cohort	374 asymptomatic high-risk women at 13–24 weeks and CL ≤ 15 mm—129 Rx and 245 not Rx. Cervical cerclage performed on >60% of cases and vaginal progesterone given to most.	Azithromycin—IV or oral or both for 7 days.	SPTB rates—51.2% vs. 50.6% in the azithromycin and untreated groups. No difference in SPTB <28, <34 weeks and PPROM. **Conclusion:** The data do not support the routine use of azithromycin in women with a short cervix and AFS.
Yeo et al., 2022 [73]	Case report	Symptomatic woman presenting at 20^+6^ weeks and sludge—treatment started at 22 weeks.	Short cervix, amniocentesis (sterile inflammation), 11 days’ treatment with IV ceftriaxone (1 gm daily), IV metronidazole 500 mg 8 hourly, and oral clarithromycin 500 mg 12 hourly.	Short cervix progressively became normal and sludge disappeared. Elective delivery at 36^+2^ weeks.

AFS—amniotic fluid sludge; PPROM = preterm prelabour rupture of fetal membranes; SPTB = spontaneous preterm birth; Rx = treatment; IV = intravenous.

## References

[1] Blencowe H., Cousens S., Chou D., Oestergaard M., Say L., Moller A.-B., Kinney M., Lawn J., on behalf of the Born Too Soon Preterm Birth Action Group (2013). Born too soon: The global epidemiology of 15 million preterm births. Reprod. Health.

[2] World Health Organization (2020). Children: Improving Survival and Well-Being.

[3] Ahmed B., Abushama M., Konje J.C. (2023). Prevention of spontaneous preterm delivery—An update on where we are today. J. Matern. Fetal Neonatal Med..

[4] Chawanpaiboon S., Vogel J.P., Moller A.-B., Lumbiganon P., Petzold M., Hogan D., Landoulsi S., Jampathong N., Kongwattanakul K., Laopaiboon M. (2019). Global, regional, and national estimates of levels of preterm birth in 2014: A systematic review and modelling analysis. Lancet Glob. Health.

[5] Ohuma E.O., Moller A.B., Bradley E., Chakwera S., Hussain-Alkhateeb L., Lewin A., Okwaraji Y.B., Mahanani W.R., Johansson E.W., Lavin T. (2023). National, regional, and global estimates of preterm birth in 2020, with trends from 2010: A systematic analysis. Lancet.

[6] Muglia L.J., Katz M. (2010). The enigma of spontaneous preterm birth. N. Engl. J. Med..

[7] Cavoretto P.I., Candiani M., Farina A. (2024). Spontaneous Preterm Birth Phenotyping Based on Cervical Length and Immune-Mediated Factors. JAMA Netw. Open.

[8] Villar J., Cavoretto P.I., Barros F.C., Romero R., Papageorghiou A.T., Kennedy S.H. (2024). Etiologically Based Functional Taxonomy of the Preterm Birth Syndrome. Clin. Perinatol..

[9] Villar J., Restrepo-Méndez M.C., McGready R., Barros F.C., Victora C.G., Munim S., Papageorghiou A.T., Ochieng R., Craik R., Barsosio H.C. (2021). Association Between Preterm-Birth Phenotypes and Differential Morbidity, Growth, and Neurodevelopment at Age 2 Years: Results From the INTERBIO-21st Newborn Study. JAMA Pediatr..

[10] Frenquelli R., Ratcliff M., Villar de Onis J., Fernandes M., Barros F.C., Hirst J.E., Papageorghiou A.T., Kennedy S.H., Villar J. (2022). Complex Perinatal Syndromes Affecting Early Human Growth and Development: Issues to Consider to Understand Their Aetiology and Postnatal Effects. Front. Neurosci..

[11] Romero R., Jung E., Chaiworapongsa T., Erez O., Gudicha D.W., Kim Y.M., Kim J.S., Kim B., Kusanovic J.P., Gotsch F. (2022). Toward a new taxonomy of obstetrical disease: Improved performance of maternal blood biomarkers for the great obstetrical syndromes when classified according to placental pathology. Am. J. Obstet Gynecol..

[12] Sovio U., Gaccioli F., Cook E., Charnock-Jones D.S., Smith G.C.S. (2024). Association between adverse pregnancy outcome and placental biomarkers in the first trimester: A prospective cohort study. BJOG.

[13] Cavoretto P.I., Farina A., Salmeri N., Syngelaki A., Tan M.Y., Nicolaides K.H. (2024). First trimester risk of preeclampsia and rate of spontaneous birth in patients without preeclampsia. Am. J. Obstet. Gynecol..

[14] Villar J., Knight H.E., de Onis M., Bertino E., Gilli G., Papageorghiou A.T., Ismail L.C., Barros F.C., Bhutta Z.A., International Fetal and Newborn Growth Consortium (INTERGROWTH-21st) (2010). Conceptual issues related to the construction of prescriptive standards for the evaluation of postnatal growth of preterm infants. Arch. Dis. Child..

[15] National Institute for Health and Care Excellence (NICE) (2015). Preterm Labour and Birth. NICE Guideline (NG25). www.nice.org.uk/guidance/ng25.

[16] Watts D.H., Krohn M.A., Hiler S.L., Eschenbach D.A. (1992). The association of occult amniotic Fluid. infection with gestational age and neonatal outcome in women in preterm labor. Obstet. Gynecol..

[17] Agrawal V., Hirsch E. (2012). Intrauterine infection and preterm labor. Semin. Fetal Neonatal Med..

[18] Romero R., Sirtori M., Oyarzun E., Avila C., Mazor M., Callahan R., Sabo V., Athanassiadis A.P., Hobbins J.C. (1989). Infection and labor. V. Prevalence, microbiology, and clinical significance of intraamniotic infection in women with preterm labor and intact membranes. Am. J. Obstet. Gynecol..

[19] Goldenberg R.L., Andrews W.W. (1996). Intrauterine infection and why preterm prevention programs have failed. Am. J. Public Health.

[20] Kim C.J., Romero R., Chaemsaithong P., Chaiyasit N., Yoon B.H., Kim Y.M. (2015). Acute chorioamnionitis and funisitis: Definition, pathologic features, and clinical significance. Am. J. Obstet. Gynecol..

[21] Romero R., Salafia C.M., Athanassiadis A.P., Hanaoka S., Mazor M., Sepulveda W., Bracken M.B. (1992). The relationship between acute inflammatory lesions of the preterm placenta and amniotic fluid microbiology. Am. J. Obstet. Gynecol..

[22] Lee S.M., Park K.H., Jung E.Y., Jang J.A., Yoo H.N. (2017). Frequency and clinical significance of short cervix in patients with preterm premature rupture of membranes. PLoS ONE.

[23] Kiefer D.G., Keeler S.M., Rust O.A., Wayock C.P., Vintzileos A.M., Hanna N. (2009). Is midtrimester short cervix a sign of intraamniotic inflammation?. Am. J. Obstet. Gynecol..

[24] Cassell G.H., Davis R.O., Waites K.B., Brown M.B., Marriott P.A., Stagno S., Davis J.K. (1983). Isolation of Mycoplasma hominis and Ureaplasma urealyticum from amniotic fluid at 16–20 weeks of gestation: Potential effect on outcome of pregnancy. Sex. Transm. Dis..

[25] Daskalakis G., Psarris A., Koutras A., Fasoulakis Z., Prokopakis I., Varthaliti A., Karasmani C., Ntounis T., Domali E., Theodora M. (2023). Maternal Infection and Preterm Birth: From Molecular Basis to Clinical Implications. Children.

[26] Romero R., Miranda J., Chaiworapongsa T., Chaemsaithong P., Gotsch F., Dong Z., Ahmed A.I., Yoon B.H., Hassan S.S., Kim C.J. (2014). A novel molecular microbiologic technique for the rapid diagnosis of microbial invasion of the amniotic cavity and intra-amniotic infection in preterm labor with intact membranes. Am. J. Reprod. Immunol..

[27] Espinoza J., Gonçalves L.F., Romero R., Nien J.K., Stites S., Kim Y.M., Hassan S., Gomez R., Yoon B.H., Chaiworapongsa T. (2005). The prevalence and clinical significance of amniotic fluid ‘sludge’ in patients with preterm labor and intact membranes. Ultrasound Obstet. Gynecol..

[28] Benacerraf B.R., Gatter M.A., Ginsburgh F. (1984). Ultrasound diagnosis of meconium-stained amniotic fluid. Am. J. Obstet. Gynecol..

[29] DeVore G.R., Platt L.D. (1986). Ultrasound appearance of particulate matter in amniotic cavity: Vernix or meconium?. J. Clin. Ultrasound.

[30] Sepulveda W.H., Quiroz V.H. (1989). Sonographic detection of echogenic amniotic fluid and its clinical significance. J. Perinat. Med..

[31] Sherer D.M., Abramowicz J.S., Smith S.A., Woods J.R. (1991). Sonographically homogeneous echogenic amniotic fluid in detecting meconium-stained amniotic fluid. Obstet. Gynecol..

[32] Vohra N., Rochelson B., Smith-Levitin M. (2003). Three-dimensional sonographic findings in congenital (harlequin) ichthyosis. J. Ultrasound Med..

[33] Bujold E., Pasquier J.C., Simoneau J., Arpin M.H., Duperron L., Morency A.M., Audibert F. (2006). Intra-amniotic sludge, short cervix, and risk of preterm delivery. J. Obstet. Gynaecol. Can..

[34] Adanir I., Ozyuncu O., Gokmen Karasu A.F., Onderoglu L.S. (2018). Amniotic fluid “sludge”; prevalence and clinical significance of it in asymptomatic patients at high risk for spontaneous preterm delivery. J. Matern. Fetal Neonatal Med..

[35] Parulekar S.G. (1983). Ultrasonographic demonstration of floating Ultrasonographic demonstration of floating particles in amniotic fluid. J. Ultrasound Med..

[36] Kusanovic J.P., Espinoza J., Romero R., Gonçalves L.F., Nien J.K., Soto E., Khalek N., Camacho N., Hendler I., Mittal P. (2007). Clinical significance of the presence of amniotic fluid ‘sludge’ in asymptomatic patients at high risk for spontaneous preterm delivery. Ultrasound Obstet. Gynecol..

[37] Bearfield C., Davenport E.S., Sivapathasundaram V., Allaker R.P. (2002). Possible association between amniotic fluid microorganism infection and microflora in the mouth. BJOG Int. J. Obstet. Gynaecol..

[38] Gomez-Lopez N., Romero R., Xu Y., Miller D., Unkel R., Shaman M., Jacques S.M., Panaitescu B., Garcia-Flores V., Hassan S.S. (2017). Neutrophil Extracellular Traps in the Amniotic Cavity of Women with Intra-Amniotic Infection: A New Mechanism of Host Defense. Reprod. Sci..

[39] Gomez-Lopez N., Romero R., Garcia-Flores V., Xu Y., Leng Y., Alhousseini A., Hassan S.S., Panaitescu B. (2017). Amniotic fluid neutrophils can phagocytize bacteria: A mechanism for microbial killing in the amniotic cavity. Am. J. Reprod. Immunol..

[40] Chen G.Y., Nunez G. (2010). Sterile inflammation: Sensing and reacting to damage. Nat. Rev. Immunol..

[41] Romero R., Miranda J., Chaiworapongsa T., Korzeniewski S.J., Chaemsaithong P., Gotsch F., Dong Z., Ahmed A.I., Yoon B.H., Hassan S.S. (2014). Prevalence and clinical significance of sterile intra-amniotic inflammation in patients with preterm labor and intact membranes. Am. J. Reprod. Immunol..

[42] Romero R., Miranda J., Chaemsaithong P., Chaiworapongsa T., Kusanovic J.P., Dong Z., Ahmed A.I., Shaman M., Lannaman K., Yoon B.H. (2014). Sterile and microbial-associated intra-amniotic inflammation in preterm prelabor rupture of membranes. J. Matern. Fetal Neonatal Med..

[43] Romero R., Miranda J., Chaiworapongsa T., Chaemsaithong P., Gotsch F., Dong Z., Ahmed A.I., Yoon B.H., Hassan S.S., Kim C.J. (2015). Sterile intra-amniotic inflammation in asymptomatic patients with a sonographic short cervix: Prevalence and clinical significance. J. Matern. Fetal Neonatal Med..

[44] Romero R., Schaudinn C., Kusanovic J.P., Gorur A., Gotsch F., Webster P., Nhan-Chang C.L., Erez O., Kim C.J., Espinoza J. (2008). Detection of a microbial biofilm in intraamniotic infection. Am. J. Obstet. Gynecol..

[45] Donlan R.M., Costerton J.W. (2002). Biofilms: Survival mechanisms of clinically relevant microorganisms. Clin. Microbiol. Rev..

[46] Costerton W., Veeh R., Shirtliff M., Pasmore M., Post C., Ehrlich G. (2003). The application of biofilm science to the study and control of chronic bacterial infections. J. Clin. Investig..

[47] Donlan R.M. (2000). Role of biofilms in antimicrobial resistance. ASAIO J..

[48] Jensen E.T., Kharazmi A., Lam K., Costerton J.W., Hoiby N. (1990). Human polymorphonuclear leukocyte response to *Pseudomonas aeruginosa* grown in biofilms. Infect. Immun..

[49] Jensen E.T., Kharazmi A., Hoiby N., Costerton J.W. (1992). Some bacterial parameters influencing the neutrophil oxidative burst response to *Pseudomonas aeruginosa* biofilms. APMIS.

[50] Wu T., Li S., Gong X., Li J., Li X., Zhai Y., Huang J., Li X., Li L., Yang J. (2024). Longitudinal cervical length measurements and spontaneous preterm birth in singleton and twin pregnancies. JAMA Netw Open..

[51] Kim C.J., Romero RChaemsaithong P., Kim J.S. (2015). Chronic inflammation of the placenta.: Definition classification, pathogenesis and clinical significance. Am. J. Obstet. Gynecol..

[52] Hatanaka A.R., Mattar R., Kawanami T.E., França M.S., Rolo L.C., Nomura R.M., Araujo Júnior E., Nardozza L.M., Moron A.F. (2016). Amniotic fluid “sludge” is an independent risk factor for preterm delivery. J. Matern. Fetal Neonatal Med..

[53] Romero R., Kusanovic J.P., Espinoza J., Gotsch F., Nhan-Chang C.L., Erez O., Kim C.J., Khalek N., Mittal P., Goncalves L.F. (2007). What is amniotic fluid sludge?. Ultrasound Obstet. Gynecol..

[54] Yoneda N., Yoneda S., Niimi H., Ito M., Fukuta K., Ueno T., Ito M., Shiozaki A., Kigawa M., Kitajima I. (2018). Sludge reflects intra-amniotic inflammation with or without microorganisms. Am. J. Reprod. Immunol..

[55] Kusanovic J.P., Jung E., Romero R., Green P.M., Nhan-Chang C.-L., Vaisbuch E., Erez O., Kim C.J., Gonçalves L.F., Espinoza J. (2022). Characterization of amniotic fluid sludge in preterm and term gestations. J. Matern. Fetal Neonatal Med..

[56] Gill N., Romero R., Pacora P., Tarca A.L., Benshalom-Tirosh N., Pacora P., Kabiri D., Tirosh D., Jung E.J., Yeo L. (2019). 467: Patients with Short Cervix and Amniotic Fluid Sludge Delivering ≤32 Weeks Have Stereotypic Inflammatory Signature. Am. J. Obstet. Gynecol..

[57] Paules C., Moreno E., Gonzales A., Fabre E., González de Agüero R., Oros D. (2016). Amniotic fluid sludge as a marker of intra-amniotic infection and histological chorioamnionitis in cervical insufficiency: A report of four cases and literature review. J. Matern. Fetal Neonatal Med..

[58] Ventura W., Nazario C., Ingar J., Huertas E., Limay O., Castillo W. (2011). Risk of impending preterm delivery associated with the presence of amniotic fluid sludge in women in preterm labor with intact membranes. Fetal Diagn. Ther..

[59] Himaya E., Rhalmi N., Girard M., Tétu A., Desgagné J., Abdous B., Gekas J., Giguère Y., Bujold E. (2011). Midtrimester intra-amniotic sludge and the risk of spontaneous preterm birth. Am. J. Perinatol..

[60] Pedregosa J.P., Ruiz C.M., Medina T.B., Rascin A.G., del Gallo J., de la Fuente J.L., Alonso M.J.T. (2017). Amniotic sludge and short cervix as inflammation and intraamniotic infection markers. Obstet. Gynecol. Int. J..

[61] Buyuk G.N., Oskovi-Kaplan Z.A., Kahyaoglu S., Engin-Ustun Y. (2021). Echogenic particles in the amniotic fluid of term low-risk pregnant women: Does it have a clinical significance?. J. Obstet. Gynaecol..

[62] Kaluarachchi A., Jayawardena G.R.M.U.G.P., Ranaweera A.K.P., Rishard M.R.M. (2018). Hyperechoic amniotic fluid in a term pregnancy. J. Fam. Med. Prim. Care.

[63] Zimmer E.Z., Bronshtein M. (1996). Ultrasonic features of intraamniotic ‘unidentified debris’ at 14–16 weeks’ gestation. Ultrasound Obstet. Gynecol..

[64] Tsunoda Y., Fukami T., Yoneyama K., Kawabata I., Takeshita T. (2020). The presence of amniotic fluid sludge in pregnant women with a short cervix: An independent risk of preterm delivery. J. Matern. Fetal Neonatal Med..

[65] Yasuda S., Tanaka M., Kyozuka H., Suzuki S., Yamaguchi A., Nomura Y., Fujimori K. (2020). Association of amniotic fluid sludge with preterm labor and histologic chorioamnionitis in pregnant Japanese women with intact membranes: A retrospective study. J. Obstet. Gynaecol. Res..

[66] Pahlavan F., Niknejad F., Irani S., Niknejadi M. (2022). Does Amniotic Fluid Sludge Result in Preterm Labor in Pregnancies after Assisted Reproduction Technology? A Nested Case—Control Study. J. Matern. Fetal Neonatal Med..

[67] Cuff R.D., Carter E., Taam R., Bruner E., Patwardhan S., Newman R.B., Chang E.Y., Sullivan S.A. (2020). Effect of Antibiotic Treatment of Amniotic Fluid Sludge. Am. J. Obstet. Gynecol. MFM.

[68] Hatanaka A.R., Franca M.S., Hamamoto T.E.N.K., Rolo L.C., Mattar R., Moron A.F. (2019). Antibiotic treatment for patients with amniotic fluid “sludge” to prevent spontaneous preterm birth: A historically controlled observational study. Acta Obstet. Gynecol. Scand..

[69] Pustotina O. (2020). Effects of antibiotic therapy in women with the amniotic fluid “sludge” at 15–24 weeks of gestation on pregnancy outcomes. J. Matern. Fetal Neonatal Med..

[70] Jin W.H., Ha Kim Y., Kim J.W., Kim T.Y., Kim A., Yang Y. (2021). Antibiotic treatment of amniotic fluid “sludge” in patients during the second or third trimester with uterine contraction. Int. J. Gynaecol. Obstet..

[71] Giles M.L., Krishnaswamy S., Metlapalli M., Roman A., Jin W., Li W., Mol B.W., Sheehan P., Said J. (2023). Azithromycin treatment for short cervix with or without amniotic fluid sludge: A matched cohort study. Aust. N. Z. J. Obstet. Gynaecol..

[72] Fuchs F., Boucoiran I., Picard A., Dube J., Wavrant S., Bujold E., Audibert F. (2015). Impact of amniotic fluid “sludge” on the risk of preterm delivery. J. Matern. Fetal Neonatal Med..

[73] Yeo L., Romero R., Chaiworapongsa T., Para R., Johnson J., Kmak D., Jung E., Yoon B.H., Hsu C.D. (2022). Resolution of acute cervical insufficiency after antibiotics in a case with amniotic fluid sludge. J. Matern. Fetal Neonatal Med..

[74] Sapantzoglou I., Pergialiotis V., Prokopakis I., Douligeris A., Stavros S., Panagopoulos P., Theodora M., Antsaklis P., Daskalakis G. (2024). Antibiotic therapy in patients with amniotic fluid sludge and risk of preterm birth: A meta-analysis. Arch. Gynecol. Obstet..

[75] Pannain G.D., Pereira A.M.G., Rocha M.L.T.L.F.D., Lopes R.G.C. (2023). Amniotic Sludge and Prematurity: Systematic Review and Meta-analysis. Rev. Bras. Ginecol. Obstet..

[76] Luca S.T., Săsăran V., Muntean M., Mărginean C. (2024). A Review of the Literature: Amniotic Fluid “Sludge”-Clinical Significance and Perinatal Outcomes. J. Clin. Med..

[77] Karampitsakos T., Mavrogianni D., Machaiotis N., Potiris A., Pangagopoulos P., Stavros S., Antsaklis P., Drakakis P. (2025). The impact of amniotic fluid interleukin-6, interleukin -8 and metalloproteinase -9 on preterm labor: A narrative reveiw. Biomedicines.

